# Influence of borderline cefepime MIC on the outcome of cefepime-susceptible *Pseudomonas aeruginosa* bacteremia treated with a maximal cefepime dose: a hospital-based retrospective study

**DOI:** 10.1186/s12941-017-0227-8

**Published:** 2017-07-24

**Authors:** Ting-Yi Su, Jung-Jr Ye, Chien-Chang Yang, Ching-Tai Huang, Ju-Hsin Chia, Ming-Hsun Lee

**Affiliations:** 1 Division of Infectious Diseases, Department of Internal Medicine, Chang Gung Memorial Hospital at Linkou, Chang Gung University College of Medicine, 5 Fu-Shin Street, Gueishan 333, Taoyuan, Taiwan; 2 Department of Laboratory Medicine, Chang Gung Memorial Hospital at Linkou, Taoyuan, Taiwan; 3 Department of Medical Biotechnology and Laboratory Science, Chang Gung University, Taoyuan, Taiwan

**Keywords:** *Pseudomonas aeruginosa*, Bacteremia, Cefepime, Minimal inhibitory concentrations, Maximal cefepime dose

## Abstract

**Background:**

We assessed the influence of current cefepime minimal inhibitory concentration (MIC) breakpoints and the maximal cefepime dose on treatment outcomes in patients with bacteremia caused by cefepime-susceptible *Pseudomonas aeruginosa*.

**Methods:**

Adult patients hospitalized between July 2010 and June 2014 with a positive blood culture for cefepime-susceptible *P. aeruginosa* and receipt of cefepime as the primary therapy throughout the course were reviewed. Cefepime Etest^®^ MICs and clinical outcomes for *P. aeruginosa* bacteremia were reviewed to identify the MIC breakpoint influencing treatment outcomes.

**Results:**

Of the 90 patients enrolled, 49 (54.4%) were male (mean age = 66.8 years). The mean Acute Physiology and Chronic Health Evaluation II score was 22.01. Sixty patients (66.7%) received a maximal cefepime dose, and the 30-day crude mortality rate was 36.7%. MIC_90_ of cefepime for *P. aeruginosa* was 8 mg/L. The cumulative survival rate at 30 days revealed that a lower cefepime MIC (<4 mg/L) for *P. aeruginosa* was associated with a higher survival rate than a higher MIC (≥4 mg/L) (72.6% vs. 23.5%, *p* < 0.0001). A cefepime MIC of ≥4 mg/L and age were independent risk factors for mortality, whereas the maximal cefepime dose was the independent protective factor. The use of a maximal cefepime dose did not improve the outcomes of patients with *P. aeruginosa* bacteremia at a MIC of ≥4 mg/L.

**Conclusions:**

A cefepime MIC of 4 mg/L may predict an unfavorable outcome among patients with serious infections caused by *P. aeruginosa*, even the MICs still within the CLSI susceptibility breakpoint.

## Background

*Pseudomonas aeruginosa* is a leading cause of nosocomial infections [1, 2], which are often life threatening [3]. Recently, actual minimal inhibitory concentrations (MICs) of fluoroquinolones [4], extended-spectrum penicillins [5], and carbapenems [6] have predicted patient outcomes more accurately than did the categorical classification of MICs as susceptible, intermediate, and resistant. Cefepime is a fourth-generation cephalosporin with a broad-spectrum antibacterial activity; it has been widely used since its approval for clinical use in 1997 [7]. According to the Clinical and Laboratory Standards Institute (CLSI) criteria of 2016 [8], the susceptible range of cefepime MIC was ≤8 mg/L. However, the mortality rates of patients infected with gram-negative organisms treated with cefepime increased with increasing MICs [9]. Therefore, the primary aim of this study was to determine the predictive value of cefepime MICs on the therapeutic outcomes in patients with cefepime-susceptible *P. aeruginosa* bacteremia and to evaluate if the current cefepime breakpoints for *P. aeruginosa* require revision.

The present recommended cefepime dosage may be suboptimal for the treatment of infections caused by *P. aeruginosa* strains with a higher cefepime MIC value [10, 11], and therapy with a higher cefepime dose was associated with a lower mortality rate in patients with gram-negative bacilli (GNB) infections [12] and requirement of the intensive care [13, 14]. The secondary aim of this study was to evaluate whether the maximal cefepime dose could improve clinical outcomes in patients with cefepime-susceptible *P. aeruginosa* bacteremia.

## Methods

### Setting

This retrospective study was conducted at the Chang Gung Memorial Hospital (CGMH), Linkou, Northern Taiwan, a 3715-bed university-affiliated tertiary-care medical center with 308 intensive care unit (ICU) beds. All clinical specimens were processed using computer-assisted microbiology laboratory databases at a central microbiology laboratory. This study was approved by the Institutional Review Board of the CGMH (103-3354B).

### Study design and patients

**Fig. 1:**
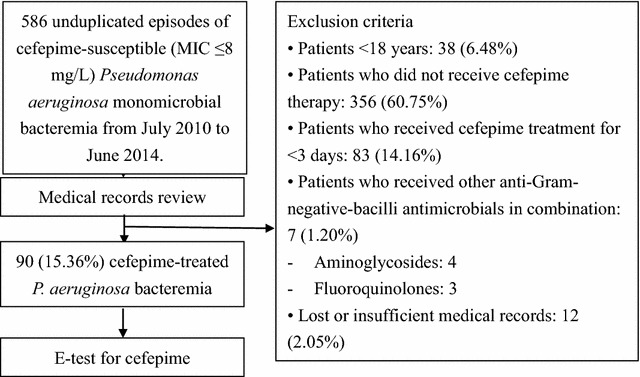
Flow chart of the exclusion of patients with *Pseudomonas aeruginosa*

In this retrospective study, 586 patients admitted to CGMH from July 2010 to June 2014 with an unduplicated monomicrobial blood culture positive for cefepime-susceptible *P. aeruginosa* and a clinical syndrome suggestive of systemic infection were reviewed. The additional inclusion criteria are as follows: age ≥18 years, clear medical records, cefepime as the first-line therapy within 48 h of bacteremia onset and monotherapy against GNB throughout the treatment. Patients who met any of the following criteria were not eligible for the study: no receipt of cefepime therapy, receipt of cefepime <3 days, combination therapy with other antimicrobials against GNB including aminoglycosides, anti-pseudomonal β-lactams or anti-pseudomonal fluoroquinolones, and inadequate clinical information from the medical records. In this study, none of the patients had received cefepime more than 3 days initially, and then received other antibiotics instead. Finally, ninety patients were enrolled in this study (Fig. 1).

### Microbiology

Blood cultures were processed in the clinical microbiology laboratory by using an automated blood culture system (BACTEC 9240 system; Becton–Dickinson Diagnostic Instrument Systems, Sparks, MD, USA). Before June 2013, *P. aeruginosa* isolates were identified on the basis of the following properties: aerobic GNB on Gram staining with glucose nonfermentation, positive oxidase test, blue–green or yellow–green fluorescent pigment production, and growth at 42 °C [15]. After June 2013, bacterial species were identified through matrix-assisted laser desorption ionization-time of flight (MALDI-TOF). *P. aeruginosa* blood isolates are routinely preserved at our clinical microbiology laboratory in skimmed milk at −70 °C until further use. All the *P. aeruginosa* blood isolates investigated in this study were selected from these stocks and tested to determine the cefepime MICs using Etest^®^ strips (bioMerieux, Lyon, France) according to the manufacturer instructions. An isolate of *P. aeruginosa* was defined as cefepime susceptible, intermediate, or resistant if its MIC was ≤8, 16, or ≥32 mg/L, respectively [8]. *P. aeruginosa* ATCC 27853 was the control.

### Data collection and definition

Demographic data, such as age, sex, concomitant diseases, and clinical characteristics, of patients with *P. aeruginosa* bacteremia were retrieved by reviewing inpatient medical records. Concomitant diseases included severe renal impairment (defined as chronic kidney disease stage 4, 5 and needed renal replacement therapy), diabetes mellitus, cerebral vascular accident, liver cirrhosis, chronic pulmonary disease, and malignancy. Central venous catheter (CVC) placement, ventilator use, ICU stay, and the time interval between hospitalization and occurrence of *P. aeruginosa* bacteremia were recorded. Disease severity scores were calculated using the Acute Physiology and Chronic Health Evaluation II (APACHE II) score on the day *P. aeruginosa* bacteremia occurred. All the patients had collected the following parameters, age, comorbidities, systolic and mean arterial blood pressure (mmHg), heart rate, respiratory rate, body temperature, initial Glasgow Coma Scale score, arterial blood gas analysis: pH, arterial oxygen tension (PaO_2_), arterial carbon dioxide tension (pCO_2_), laboratory data (white blood cell count, hematocrit, sodium, potassium, and creatinine). However, the following values, if missing, were considered normal: PaO_2_, pH, and pCO_2_. Severe sepsis were defined as sepsis plus evidence of organ dysfunction included either one criteria as bellowed: (1) arterial hypoxemia (PaO_2_/fraction of inspiration O_2_; FiO_2_ <300), (2) acute oliguria (urine output <0.5 mL/kg per hour for at least 2 h despite adequate fluid resuscitation, (3) increase in creatinine >0.5 mg/dL, (4) coagulation abnormalities: international normalized ratio (INR) >1.5, activated partial thromboplastin time (aPTT) >60 s, platelets <100,000/μL, (5) hepatic dysfunction (elevated bilirubin), (6) paralytic ileus, and (7) decreased capillary refill or skin mottling. Septic shock was defined as sepsis with hypotension refractory to fluid resuscitation or hyperlactatemia. Neutropenia was defined as absolute neutrophil count of <0.5 × 10^9^/L.

The sources of bacteremia determined from medical records, imaging studies, surgical findings, and microbiological evidences were categorized into lower respiratory and urinary tracts, skin and skin structure, central catheter-associated bloodstream infection (CABSI), and intra-abdominal infections. If no source was identified, the infection was categorized as primary bacteremia.

### Treatment and outcomes

The dosage and dosing frequency of cefepime were reviewed from patient medical records. Cefepime was infused over 30 min. Creatinine clearance (CL_CR_) was calculated on the date of first dose of cefepime was given. CL_CR_ was calculated using an adjusted Cockcroft–Gault equation that excluded patient weight [CL_CR_ = (140 − age)/serum creatinine concentration]; the result was multiplied by 0.85 for female patients. The maximal cefepime dose adjusted by CL_CR_ was defined as 2 g every 8 h, 2 g every 12 h, 2 g every 24 h, and 1 g every 24 h, while CL_CR_ was ≥50, 30–49, 10–29, and <10 mL/min, respectively [10]. Patients receiving above CL_CR_-adjusted dosing regimens throughout the course of cefepime treatment were defined as using the maximal cefepime dose. Clinical outcomes were assessed using the 30-day crude mortality.

### Statistical analyses

All statistical analyses were performed using the Statistical Package for Social Sciences for Windows (version 18.0; SPSS Inc., Chicago, IL, USA). Categorical variables were compared using the χ^2^ or Fisher exact tests, as appropriate; continuous variables were compared using the Mann–Whitney *U* test. Variables with *p* < 0.1 in the univariate analysis were included in a multiple logistic regression model using the backward stepwise method for identifying the risk factors for the 30-day sepsis-related mortality. Adjusted odds ratios (ORs) and 95% confidence intervals (CIs) were calculated. The survival curve was plotted by means of the Kaplan–Meier method, and the log rank test was used to compare univariate survival distribution. All tests were two-tailed, and *p* < 0.05 was considered significant.

## Results

### Patient enrollment and their clinical characteristics

A total of 586 unduplicated cefepime-susceptible *P. aeruginosa* blood isolates from 586 patients were identified. On the basis of our inclusion criteria, 496 patients were excluded because of age <18 years, no receipt of cefepime therapy or cefepime use <3 days, or receipt of combination therapy with other anti-GNB antimicrobials. Ninety patients with individual unduplicated *P. aeruginosa* blood isolates were enrolled (Fig. 1). Patient demographics and clinical characteristics are listed in Table 1. Of the 90 patients, 49 (54.4%) were male with a mean age of 66.8 years. The most common concomitant disease was solid organ malignancy (43.3%), followed by diabetes mellitus (32.2%), and chronic kidney disease stage IV and above (31.1%). Time between hospital admission and occurrence of *P. aeruginosa* bacteremia ranged from 0 to 252 days with a mean interval of 23.2 days.

**Table 1:** Clinical characteristics of 90 patients with cefepime-susceptible *Pseudomonas aeruginosa* monomicrobial bacteremia receiving cefepime monotherapy

Variables	Value^a^
Demographic parameters	
Age, year	66.8 (14.6)
Male gender	49 (54.4)
Concomitant diseases	
Diabetes mellitus	29 (32.2)
Severe renal impairment	28 (31.1)
Liver cirrhosis	10 (11.1)
Chronic pulmonary disease	8 (8.9)
Cerebral vascular accident	19 (21.1)
Solid organ malignancy	39 (43.3)
Haematological malignancy	16 (17.8)
Autoimmune disease	5 (5.6)
Clinical conditions	
Time interval between admission and occurrence of bacteremia, day	23.2 (36.3)
Central venous catheter use	68 (75.6)
Patients’ severity	
APACHE II score	22.07 (6.0)
Ventilator use	23 (25.6)
Intensive care unit stay	29 (32.2)
Severe sepsis or septic shock	21 (23.3)
Neutropenia	18 (20.0)
Source of bacteremia	
Primary bacteremia	38 (42.2)
Lower respiratory tract	27 (30)
Urinary tract	7 (7.8)
Skin and skin structure	2 (2.2)
Central catheter associated blood stream infection	12 (13.3)
Intra-abdominal infection	10 (11.1)
Treatment	
Use of maximum cefepime dose	60 (66.7)
Treatment duration	16.4 (7.031)
Remove catheter or operation	8 (8.9)
Outcome	
30-day crude mortality	33 (36.7)

*APACHE II score* Acute Physiology and Chronic Health Evaluation II score

^a^Categorical data: number (%) of patients; continuous data are expressed as mean (standard deviation)

Sixty-eight patients (75.6%) had received a CVC placement, 23 (25.6%) ever used a ventilator, 29 (32.2%) had ICU stay, 21 (23.3%) had severe sepsis or septic shock, and 18 (20%) had neutropenia. The mean APACHE II score was 22.01. Thirty-eight patients (38.8%) had primary bacteremia and the remaining 52 (61.2%) had identified sources of bacteremia. One case had vertebral osteomyelitis. The most common source of bacteremia was lower respiratory tract infection (27/52, 51.9%), followed by CABSI (12/52, 23.1%).

### Treatment and outcomes

Sixty patients (66.7%) used the maximal cefepime dose. The treatment duration varied from 3 to 30 days with a mean duration of 16.4 days, and the 30-day crude mortality rate was 36.7%. Besides, none of the studied cases had reported the adverse effects including neurotoxicity during cefepime use.

### MIC versus mortality

**Fig. 2:**
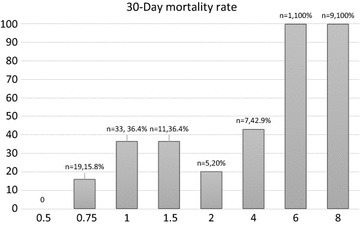
Cefepime minimal inhibitory concentrations versus rate of mortality. *n* numbers, presented as the blood isolate number and the following is mortality rate, *MIC* minimal inhibitory concentration (mg/L)

Figure 2 depicts the relationship between MICs and mortality rates. Cefepime MIC_50_ and MIC_90_ for *P. aeruginosa* were 1 and 8 mg/L, respectively. The lower MICs (0.5, 0.75, and 1 mg/L) were associated with the lower mortality rates (0, 15.8, and 36.4%, respectively). The mortality rate extended to 42.9 and 100% at the MICs of 4 and >4 mg/L, respectively.

### Risk factors for 30-day mortality of *P. aeruginosa* bacteremia

**Fig. 3:**
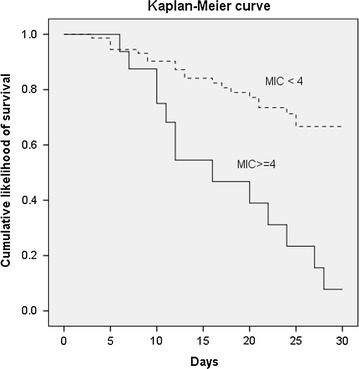
Kaplan-Meier survival curve in patients with cefepime-susceptible *Pseudomonas aeruginosa* bacteremia. Comparison of the cumulative survival between cefepime minimal inhibitory concentration (MIC) <4 and ≥4 mg/L (*p* < 0.0001)

The cumulative survival rate at 30 days revealed that a lower cefepime MIC (<4 mg/L) for *P. aeruginosa* was associated with a significantly higher survival rate than a higher MIC (≥4 mg/L) (72.6% versus 23.5%, *p* < 0.0001) (Fig. 3).

The factors associated with the 30-day mortality in univariate analysis (Table 2) included older ages (71.5 ± 12.7 vs. 64.1 ± 15.1 years, *p* = 0.028), a longer time interval between the dates of admission and positive blood cultures (31.8 ± 34.6 vs. 18.3 ± 33.6 days, *p* = 0.02), a longer ICU stay (54.5% vs. 19.3%, *p* = 0.001), more episodes of severe sepsis or septic shock (36.4% vs. 15.8%, *p* = 0.038), more respiratory tract infections (48.5% vs. 19.3%, *p* = 0.004), a higher cefepime MIC (≥4 mg/L) (76.5% vs. 27.4%, *p* < 0.001), and fewer instances of maximal cefepime dose use (48.5% vs. 78.9%, *p* = 0.003). The mortality rate of patients with a cefepime MIC of ≥4 mg/L for *P. aeruginosa* was 76.5%, which was higher than those with a MIC of <4 mg/L (27.4%).

**Table 2:** Univariate analyses of risk factors for 30-day crude mortality of cefepime-susceptible *Pseudomonas aeruginosa* bacteremia treated with cefepime

Variables	Deceased^a^	Survived^a^	Univariate
	*n* = 33	*n* = 57	*p*
Demographic parameters			
Age, year	71.5 (12.7)	64.1 (15.1)	0.028
Male gender	21 (63.6)	28 (49.1)	0.183
Concomitant diseases			
Diabetes mellitus	9 (27.3)	20 (35.1)	0.445
Severe renal impairment	13 (39.4)	15 (26.3)	0.197
Liver cirrhosis	4 (12.1)	6 (10.5)	1.000
Chronic pulmonary disease	4 (12.1)	4 (7.0)	0.458
Cerebral vascular accident	8 (24.2)	11 (19.3)	0.580
Solid organ malignancy	17 (51.5)	22 (38.6)	0.233
Haematological malignancy	4 (12.1)	12 (21.1)	0.394
Autoimmune disease	2 (6.1)	3 (5.3)	1.000
Clinical conditions			
Time interval between admission and occurrence of bacteremia, day	31.8 (34.6)	18.3 (33.6)	0.019
Central venous catheter use	27 (81.8)	41 (71.9)	0.293
Patients’ severity			
APACHE II score	23.9 (5.5)	21.0 (6.1)	0.091
Intensive care unit stay	18 (54.5)	11 (19.3)	0.001
Severe sepsis or septic shock	12 (36.4)	9 (15.8)	0.038
Neutropenia	7 (21.2)	11 (19.3)	0.827
Source of bacteremia			
Primary bacteremia	11 (33.3)	27 (47.4)	0.194
Lower respiratory tract	16 (48.5)	11 (19.3)	0.004
Urinary tract	1 (3.0)	6 (10.5)	0.416
Skin and skin structure	1 (3.0)	1 (1.8)	1.000
CABSI	3 (9.1)	9 (15.8)	0.524
Intra-abdominal site	5 (15.2)	5 (8.8)	0.488
Microbiology			
MIC ≥4 mg/L	13 (39.4)	4 (7.0)	<0.001
Treatment			
Use of maximum cefepime dose	15 (45.5)	45 (78.9)	0.001
Treatment duration	14.7 (7.2)	17.5 (6.8)	0.062
Remove catheter or operation	2 (6.1)	6 (10.5)	0.705

*APACHE II score* Acute Physiology and Chronic Health Evaluation II score, *CABSI* central catheter associated blood stream infection, *MIC* minimal inhibitory concentration

^a^Categorical data: number (%) of patients; continuous data are expressed as mean (standard deviation)

Those factors associated with the 30-day mortality in univariate analysis and APACHE II score were entered into multivariate analysis (Table 3), and the result showed that MIC ≥4 mg/L (adjusted OR 5.111; 95% CI 1.090–23.974; *p* = 0.039) and age (adjusted OR 1.065; 95% CI 1.011–1.122; *p* = 0.023) were the independent risk factors for the 30-day mortality. Maximal cefepime dose usage was an independent protecting factor (adjusted OR 0.271; 95% CI 0.08–0.889; *p* = 0.031).

**Table 3:** Multivariate analyses of risk factors for 30-day crude mortality of cefepime-susceptible *Pseudomonas aeruginosa* bacteremia treated with cefepime

Variables	*p*	OR	95% CI
MIC ≥4 mg/L	0.039	5.111	1.090–23.974
Use of maximum cefepime dose	0.031	0.271	0.082–0.889
Age, year	0.023	1.065	1.011–1.122
Lower respiratory tract infections	0.056	4.008	0.967–16.621
APACHE II score	0.824	0.986	0.869–1.119
Intensive care unit stay^a^	0.146	2.945	0.687–12.619
Severe sepsis or septic shock^a^	0.210	2.609	0.582–11.706
Time interval between admission and occurrence of bacteremia	0.861	1.002	0.983–1.021
Treatment duration	0.063	0.923	0.848–1.004

All variables with *p* < 0.1 in univariate analysis were included in a multivariate regression model using the backward stepwise method

*OR* odds ratio, *CI* confidence interval, *MIC* minimal inhibitory concentration

^a^The factors of intensive care unit stay and severe sepsis or septic shock had strongly correlation (correlation coefficient 0.9), however, single factor with either intensive care unit stay or severe sepsis and septic shock were still remained insignificantly in the multivariate analyses model (factor with intensive care unit only: adjusted OR 3.127, 95% CI 0.746–13.111, *p* = 0.119; factor with severe sepsis or septic shock only: adjusted OR 2.813, 95% CI 0.647–12.226, *p* = 0.168)

### Relationship between MIC and the maximal dose of cefepime

Seventy-three patients had *P. aeruginosa* blood isolates with a cefepime MIC of <4 mg/L. Among them, compared with the survived, the deceased had fewer patients receiving a maximal dose of cefepime (50.0% vs. 81.1%, *p* = 0.008), more solid organ malignancy (65% vs 35.8%, *p* = 0.025) and a shorter treatment duration (14.0 ± 7.0 vs. 17. 8 ± 6.6 days, *p* = 0.048). In the multivariate analysis, use of the maximal cefepime dose was the only protecting factor for mortality (adjusted OR 0.244; 95% CI 0.077–0.771; *p* = 0.016). This protection was not found among patients with a MIC of ≥4 mg/L for *P. aeruginosa*. When the MIC was ≥4 mg/L, the mortality rate of patients using the maximal cefepime dose was 75% (6 of 8 patients), which is similar to those using a lower dose of cefepime (7 of 9 patients, 77.8%, *p* = 1.000). For those patients receiving a maximal dose of cefepime (n = 60), patients with a cefepime MIC of ≥4 mg/L for *P. aeruginosa* had a higher 30-day crude mortality rate than those with a MIC of <4 mg/L (33.3% vs. 4.4%, *p* = 0.008).

## Discussion

According to our review of relevant literature, our study is the first one to provide clinical data demonstrating that treatment of cefepime-susceptible *P. aeruginosa* bacteremia with a maximal dose of cefepime improved the outcomes of patients with a lower cefepime MIC for *P. aeruginosa*. Besides, the current CLSI criteria for cefepime susceptibility did not predict clinical outcomes appropriately in this study. The 30-day crude mortality rate was 36.7% and the mortality rate was higher at the group of patients with a MIC of ≥4 mg/L for *P. aeruginosa* than those with a MIC of <4 mg/L (76.5% vs. 27.4%). Cefepime MIC ≥4 mg/L influenced patient outcomes independently, whereas using a maximal dose of cefepime in patients with various degrees of renal function was the only independent protecting factor for mortality. In addition, using the maximal dose of cefepime significantly decreased the mortality rate at patients with a MIC of <4 mg/L for *P. aeruginosa*. However, the protective effect vanished at a MIC of ≥4 mg/L. Our results revealed that using the maximal cefepime dose could improve patient outcomes at a lower MIC level. In this study, the antibiotic susceptibility testing was performed using Etest, not broth microdilution (BMD) methods, which is the CLSI criteria based on. However, Etest results generally have correlated well with MICs generated by BMD method [16]. Thus, the current CLSI criteria for cefepime susceptibility breakpoint of ≤8 mg/L may be reevaluated for severe *P. aeruginosa* infections.

In optimal situations, antibiotic susceptibility breakpoints are determined by integrating various microbiologic, pharmacokinetic/pharmacodynamic (PK/PD), and clinical data. However, after antibiotics were released commercially, new mechanisms of antibiotic resistance developed and probably affected the efficacy of antibiotics. Falagas et al. [17] described that high MICs of GNB, particularly in *Salmonella enterica* and *P. aeruginosa* infections, within the currently accepted “susceptible” range were associated with worse outcomes. Several studies have revealed that high piperacillin MICs are associated with increasing mortality rates and microbiological treatment failure. This led to lowering of the CLSI recommendation of the breakpoint of piperacillin against *P. aeruginosa* from ≤64 to ≤16 mg/L [5, 18, 19]. Worse outcomes related to high MICs were also found on carbapenem use for patients with either bloodstream [6] or lower respiratory tract infections [20]. Patients with levofloxacin-treated gram-negative bloodstream infections, who have elevated levofloxacin MICs but are nevertheless categorized as susceptible, had worse outcomes than those infected with gram-negative organisms, which had lower MICs [4]. Cefepime was inferior to carbapenems in treating patients with bacteremia caused by cefepime-susceptible extended-spectrum ß-lactamase producing strains. The mortality rate increased significantly because cefepime MICs increased (*p* = 0.004) [21]. Compared with a cefepime MIC of ≤4 mg/L for *P. aeruginosa*, patients with a MIC of 8 mg/L had a significantly higher mortality rate (66.7% versus 20.8%, *p* = 0.01) regardless of the cefepime dosage [9].

Studies have demonstrated that free or nonprotein-bound drug concentration over the MIC of the organism (*fT* > MIC) is the ideal predictor for bactericidal and microbiologic response for β-lactams. A larger *fT* > MIC (50–70%) is required for the maximal activity against gram-negative organisms [22]. However, several studies have now assessed the PK/PD profile of cefepime and support a change in cefepime dose or breakpoints for susceptibility. Crandon et al. [10] revealed that at the CLSI MIC breakpoint of cefepime susceptibility for *P. aeruginosa* (≤8 mg/L), a dose of only 2 g every 8 h has a ≥82% likelihood of achieving at least 60% *fT* > MIC in patients with normal renal function. At this MIC (≤8 mg/L), the dose of 1 or 2 g every 12 h for immunocompetent patients with severe *P. aeruginosa* infections has a target attainment rate of only 47.7 or 65.8%, respectively. Another PK/PD study of cefepime revealed that when *C*_67%_ >MIC was used as the pharmacodynamic target, a dose of 2 g every 12 h had a more than 80% likelihood of achieving the optimal target with an MIC of up to 4 mg/L, whereas a dose of 2 g every 24 h can probably achieve a target attainment rate of up to 80% only when the MICs were ≤2 mg/L [11]. The aforementioned studies explain the failure of achieving pharmacodynamics and the possible microbiological failure in cefepime-treated *P. aeruginosa* infections with a high cefepime MIC. In addition, they revealed the influence of different cefepime dosages on pharmacodynamics.

Alves et al. [12] demonstrated that treatment with cefepime at a dose of 2 g every 8 h over a 30-min infusion was associated with significantly lower hospital mortality rates in patients with GNB bloodstream infection compared with the usual dosage regimens, such as 1 or 2 g every 12 h and 1 g every 8 h. Moreover, they included 113 patients with *Escherichia coli* (62, 54.9%) and *P. aeruginosa* (19, 16.8%) infections. The median MIC of all GNB was 0.0625 mg/L, and most (78.8%) MICs were ≤0.25 mg/L; MIC_90_ was 2 mg/L. High-dose cefepime therapy was associated with lower mortality rates in patients with GNB infection, including GNB with a low cefepime MIC.

Our study has the limitations for being a retrospective design with the treatment decisions dependent on the physicians’ judgments and the hospital antimicrobial stewardship program [23]. However, some study results suggested E test provides equal or more clear and accurate results in clinical set-up [24, 25]. Furthermore, the mechanisms for increasing cefepime MICs in *P. aeruginosa* isolates remain unclear. Additional investigations concerning the resistance are thus necessary.

## Conclusions

In summary, our data showed that patients treated with cefepime for cefepime-susceptible *P. aeruginosa* bloodstream infections had a worse outcome while the isolates had a higher MIC value that was still within the susceptible category. Use of a higher cefepime dose in cases with a MIC of <4 mg/L for *P. aeruginosa* improved patient outcomes. Mortality rate increased in patients with a higher cefepime MIC (≥4 mg/L) for *P. aeruginosa* even with a maximal cefepime dose. Thus, when using cefepime to treat serious *P. aeruginosa* infections, the current CLSI cefepime MIC of 8 mg/L as the susceptibility breakpoint may not predict the clinical outcome well.

## Abbreviations

APACHE II: Acute Physiology and Chronic Health Evaluation II
aPTT: activated partial thromboplastin time
BMD: broth microdilution
CABSI: central catheter-associated bloodstream infection
CGMH: Chang Gung Memorial Hospital
CIs: confidence intervals
CL_CR_: creatinine clearance
CLSI: Clinical and Laboratory Standards Institute
CVC: central venous catheter
GNB: gram-negative bacilli
FiO_2_: fraction of inspiration O_2_
ICU: intensive care unit
INR: international normalized ratio
MALDI-TOF: matrix-assisted laser desorption ionization-time of flight
MICs: minimal inhibitory concentrations
ORs: odds ratios
PaO_2_: arterial oxygen tension
pCO_2_: arterial carbon dioxide tension
PK/PD: pharmacokinetic/pharmacodynamic
